# Super Resolution Imaging of Genetically Labeled Synapses in *Drosophila* Brain Tissue

**DOI:** 10.3389/fncel.2016.00142

**Published:** 2016-05-26

**Authors:** Isabelle A. Spühler, Gaurasundar M. Conley, Frank Scheffold, Simon G. Sprecher

**Affiliations:** ^1^Department of Physics, University of FribourgFribourg, Switzerland; ^2^Department of Biology, University of FribourgFribourg, Switzerland

**Keywords:** synapses, brain tissue, olfactory projection neurons, mushroom body, *d*STORM

## Abstract

Understanding synaptic connectivity and plasticity within brain circuits and their relationship to learning and behavior is a fundamental quest in neuroscience. Visualizing the fine details of synapses using optical microscopy remains however a major technical challenge. Super resolution microscopy opens the possibility to reveal molecular features of synapses beyond the diffraction limit. With direct stochastic optical reconstruction microscopy, *d*STORM, we image synaptic proteins in the brain tissue of the fruit fly, *Drosophila melanogaster*. Super resolution imaging of brain tissue harbors difficulties due to light scattering and the density of signals. In order to reduce out of focus signal, we take advantage of the genetic tools available in the *Drosophila* and have fluorescently tagged synaptic proteins expressed in only a small number of neurons. These neurons form synapses within the calyx of the mushroom body, a distinct brain region involved in associative memory formation. Our results show that super resolution microscopy, in combination with genetically labeled synaptic proteins, is a powerful tool to investigate synapses in a quantitative fashion providing an entry point for studies on synaptic plasticity during learning and memory formation.

## Introduction

In recent years, an increasing effort has been put toward the characterization of neuronal connectivity on large scales (Lichtman and Denk, 2011; Aso et al., 2014; Shih et al., 2015), as well as toward a better understanding of plasticity of synaptic structures in relation to learning and memory formation (Kremer et al., 2010). However, investigating the fine structure and molecular architecture of synapses in brain tissue bears major technical difficulties. Optical microscopy allows to examine multiple proteins using various fluorescent markers, but optical diffraction prevents access to nanoscale characteristics of synaptic connections between neurons. The latter have so far been studied mainly with electron microscopy (EM), which demands a compromise between the quality of the preserved ultrastructure and the depth of antibody penetration for labeling (Lakadamyali, 2012; Ehmann et al., 2015). Super resolution microscopy allows us to overcome these technical limits and enables high resolution imaging of various, specifically labeled proteins. Among the different methods of super resolution microscopy, single molecule localization microscopy (SMLM) has proven to be a versatile and reliable method to image and quantify subcellular structures (Specht et al., 2013; Ehmann et al., 2014). Imaging with SMLM relies on the sequential activation and localization of single fluorescent molecules, whose positions are then used to reconstruct a super resolution image. So far it has been applied mainly on cells in culture and subcellular structures such as the cytoskeleton or nuclear pore complexes (Bates et al., 2007; Szymborska et al., 2013; Xu et al., 2013). Relatively few have applied this revolutionary imaging technique on brain tissue to study the fine structure and molecular architecture of synapses (Dani et al., 2010; Beaudoin et al., 2012; Ehmann et al., 2014; Dudok et al., 2015). Reasons for this are challenges inherent to tissue imaging such as strong background signal given by out of focus fluorescence and density of labeled structures as well as unavoidable scattering of excitation and emission light, resulting in poor signal to noise ratio, and ultimately in a lower achievable resolution (Kamiyama and Huang, 2012).

In the current study, we report technical advances in the experimental approach to imaging synapses in brain sections. We use genetic tools available in the fruit fly *Drosophila melanogaster*, which allows us to confine the expression of fluorescently tagged proteins to a small subset of neurons, significantly reducing the signal density within the tissue. Furthermore, highly inclined illumination and adaptive optics are combined to overcome the aforementioned limitations to SMLM imaging of thick tissue samples. We image synaptic proteins in brain tissue sections using direct stochastic optical reconstruction microscopy, *d*STORM (Heilemann et al., 2008), a SMLM technique, which relies on stochastic blinking of conventional fluorophores. After imaging, we analyze the super resolution images to identify cluster formation and nanoscopic localization patterns. Moreover, we localize the signals within a depth of 1–3 μm and reconstruct three dimensional images based on the method of astigmatism (Huang et al., 2008b).

While confocal imaging gives coarse information about densely packed proteins, with *d*STORM we are able to resolve nanoscale spatial organization and obtain results with a resolution approaching those reported in EM studies. SMLM additionally provides the specificity of fluorescent labeling required to examine the density and distribution of specific proteins with defined function. In this way, SMLM applied on tissue section has the potential to reveal the composition and fine structure of synapses within neuronal circuits and to examine eventually structural plasticity related to learning and memory.

## Materials and methods

### Fly stock

Fly strains are reared on standard Drosophila medium at 25 or 18°C with a 14/10 h light/dark cycle. To visualize synaptic proteins in specific neurons we cross the GAL4-driver lines Mz19-Gal4/+ or 201y-Gal4 with UAS-syt::GFP, UAS-brp-short^cherry^, UAS-Dα7::GFP, UAS-brp-short^cherry^ MB247-Dα7-GFP/+ (kindly provided by G. Tavosanis, DZNE Bonn, Germany), and UAS-Drep-2 mStrawberry (kindly provided by S. Sigirst, FU Berlin, Germany).

### Tissue preparation for imaging

Adult female flies are cooled to immobility and groups of five or six flies are threaded by their necks into collar holders. Flies are fixed in 4% paraformaldehyde in 0.1 M phosphate buffered saline (PBS) for 1 h at room temperature or for 4 h at 4°C. After washing three times 10 min with PBS the flies are immersed overnight with 20% sucrose in 0.1 M PBS for cryoprotection. Eight micrometers thin cryosections are cut on a Leica 2020 vibratome and mounted on Poly-L-Lysine coated coverslips (neuVitro, #1.5 thickness).

### Immunostaining

Cryosections are first dried on coverslips for 30 min. The sections are then blocked for 30 min with 5% Normal Goat Serum in PBT (PBS containing 0.1% Triton X-100) before they are incubated for 2 h with primary antibody in blocking solution. Primary antibodies used in this study include mouse antibody to GFP (Invitrogen) and rabbit antibody to DsRed (Clontech). Sections are washed three times for 10 min with PBS and then incubated for 1 h with the secondary antibody. Secondary antibodies applied are anti-mouse or anti-rabbit Alexa Fluor 647 antibodies (Invitrogen). After final washing of three times for 10 min with PBS, coverslips are mounted on slides with a freshly prepared imaging medium. The medium contains 100 mM cysteamine, 10% (m/v) glucose, 1 mg ml^−1^ Glucose oxidase, Catalase (2.5 μl ml^−1^ of aqueous solution, all from Sigma-Aldrich). The coverslips are sealed with nail polish or Secure Seal imaging spacer (Sigma-Aldrich).

### Image acquisition and reconstruction

Super resolution and confocal imaging is performed on a Nikon Ti Eclipse inverted microscope, equipped with an A1 Confocal Scan Head, an oil immersion objective (CFI Apo TIRF 100x, NA 1.49), and a TIRF-arm for highly inclined illumination. For super resolution imaging specifically a powerful red laser (Coherent Genesis MX STM, 1 W at 639 nm) and a weaker violet one (Toptica iBeam smart, 120 mW at 405 nm) are coupled into a single mode fiber through an AOTF (Acousto-Optical Tunable Filter), which also allows for intensity control. The fiber directs the light to the TIRF-arm through which collimation and angle of illumination can be controlled. The emitted fluorescence is directed through an adaptive optics system (MicAO 3D-SR), which corrects unwanted aberrations and adds astigmatism for 3D imaging. The light is finally captured on an Andor iXon Ultra 897 EMCCD camera. The pixel size is 104 nm, achieved with extra magnification.

We first take a stack of confocal images of the region of interest with a step size of 0.2 μm. We then switch to weak wide-field illumination with the red laser and, once the plane of interest is identified, the laser is set to full power. Initially the angle of illumination is kept at 0° in order to render dark the whole thickness of the sample. After a few seconds the angle is increased to near TIRF (total internal reflection fluorescence) conditions, thereby reducing out of focus fluorescence and increasing local intensity. Once stable blinking conditions are reached, we adjust the camera integration time between 6 and 30 ms and acquire 60–100 thousand frames. During acquisition, the violet laser is sometimes used to adjust the density of blinking events.

Single fluorophores are localized and the image reconstructed with *ThunderSTORM* (Ovesny et al., 2014). For visualization we use the method of average shifted histogram reconstruction. The reconstruction is subsequently corrected for drift and adjusted for the different parameters to minimize out of focus signals.

### Image analysis for objects and clusters

Once the super resolution image is reconstructed, it can be analyzed to reveal protein organization within subcellular structures. To achieve this, we apply the recently published *SR-Tesseler* Method (Levet et al., 2015), which allows the analysis of protein organization at different scales. In this method, neighborhood relationships among signals as well as local densities are determined by segmentation of the image through Voronoï tessellation. Setting thresholds for local density and minimal area results in the identification of objects and of clusters within them. Objects and clusters can represent different subcellular structures depending on the imaged protein. To initially define the threshold for local density and minimal area, automatic thresholding as discussed in Levet et al. is applied and slightly adjusted by comparing *SR-Tesseler* image segmentations with the underlying super resolution images. We then apply the same threshold for all the four proteins in order to avoid user bias. Objects are defined as having twice the average local density and a minimal area of 0.03 μm^2^. To identify clusters formed within the objects, the threshold is set to 1.8 times the average local density of the object and 0.0005 μm^2^ as the minimal area. For quantitative comparison, the distribution of object and cluster sizes are plotted in normalized histograms and tested for statistical significance.

### 3D imaging and visualization

Some samples are also imaged to reconstruct the localization of the fluorophores within a limited depth of the tissue. For the three dimensional imaging we introduce astigmatism (MicAO 3D-SR). With the *MicAO* optic system in place the point spread function is set to change its shape depending on the axial position of the fluorescent signal relative to the focal plane. This is calibrated for using fluorescent TetraSpeck™ Microspheres (100 nm diameter, ThermoFisher Scientific), which are imaged at different axial positions. All z-coordinates are rescaled by a factor of 0.72 due to the refractive index mismatch between glass and the sample in the buffer solution (Huang et al., 2008a) and in *ThunderSTORM* the signal was adjusted for drift and noise. The three dimensional images were subsequently visualized with *ViSP* (El Beheiry and Dahan, 2013). *ViSP* further provides the possibility to define the surface area and volume of defined objects.

## Results

### Imaging active zones of olfactory projection neurons

**Figure 1:**
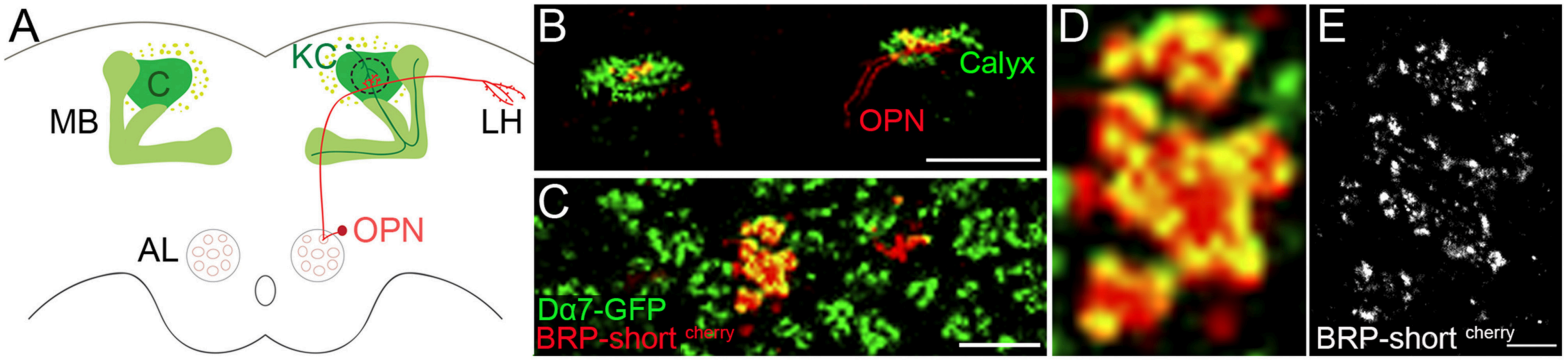
**Expression of fluorescently tagged synaptic proteins in sparse number of neurons of the olfactory circuit**. Olfactory information is sent via the olfactory projection neuron (OPN) from the antennal lobe (AL) to the mushroom body (MB). **(A)** Sketch of the OPN forming boutons in the calyx of the MB and further in the lateral horn (LH). In the current study we examine the expression of synaptic proteins in the boutons of the OPNs and the dendrites of the Kenyon cells (KC) within the calyx (outlined with dotted line). **(B)** Confocal image of BRP-short^cherry^ expressed in Mz-19 Gal4 fly line with an additional expression of Dα7 tagged with GFP in KCs, revealing the calyx of the MB. Scale Bar: 100 μm **(C)** Enlarged view on calyx of the same image shows the sparse expression of BRP-short^cherry^ and the microglomeruli characteristic synaptic complex of the calyx. Scale Bar: 10 μm **(D)** Zoom in confocal image on the microglomeruli shows the overlapping signal of the BRP-short^cherry^ additionally stained with AF647 and Dα7-GFP. **(E)** Same site imaged with *d*STORM reveals the different location of the AF647 fluorophores bound to BRP-short^cherry^ and indirectly the expression pattern of BRP in the bouton of OPN. Dense and confined signal indicates potential presynaptic sites. Scale bar **(D)** and **(E)**: 1 μm.

To study protein organization of chemical synapses in the brain we investigated synapses formed between olfactory projection neurons (OPN) and Kenyon cells (KC) within the calyx of the fruit fly. KCs receive olfactory information through OPN from the antennal lobe (Figure 1A) and it has been shown that they are involved in associative memory formation (Kremer et al., 2010). The boutons of the OPN are among the biggest in the fly brain and each axon forms multiple, prominent synapses with claw-like dendrites of KC. These synaptic complexes between OPN and KC are called microglomeruli.

To reduce the noise from out of focus signal for *d*STORM imaging in tissue, fluorescently tagged synaptic proteins are expressed in a few defined neurons. By using the MZ19-Gal4 driver, tagged presynaptic proteins are expressed only in 10–13 OPNs (Kremer et al., 2010). These neurons send their axons from the antennal lobe to the calyx of the mushroom body and to the lateral horn (Figure 1). To illustrate the microglomeruli within the calyx, the postsynaptic membrane of the KC is shown here with the expression of GFP tagged acetylcholine receptor Dα7 (UAS-brp-short^cherry^ MB247-Dα7-GFP/+ crossed with Mz19-Gal4). A subset of the MZ19 positive boutons is first imaged by confocal and subsequently with *d*STORM. Figures 1B–D show the specific expression of Bruchpilot (BRP) fluorescently tagged with mCherry (BRP-short^cherry^) under the control of MZ19-Gal4. BRP is localized at T-bar formations within the active zone and is widely used as a presynaptic marker. In earlier studies, it has been tested that the expression of UAS-brp-short^cherry^ does not affect the number of active zones (Kremer et al., 2010) and with immuno-EM it has been shown that BRP-short localizes to synaptic active zones in the fly CNS (Mosca and Luo, 2014). For *d*STORM imaging, BRP-short^cherry^ is additionally stained with Alexa Fluor 647 (AF647). On the confocal images the red presynaptic and green postsynaptic signal overlap and result in a diffuse, yellow signal (Figure 1D). The super resolution image we obtain after *d*STORM imaging depicts signals only related to BRP-short^cherry^ expression. We observe signals confined within small regions indicating potential active zones with T-bar formation in the presynaptic membrane (Figure 1E). While distinct active zones cannot always be easily distinguished in the confocal, single sites with signals related to BRP can be recognized accurately in the super resolution image.

### Comparison of different synaptic proteins for local density and distribution

SMLM enables imaging beyond the diffraction limit and thus offers potential to unravel nanoscopic features in density and distribution of different synaptic proteins. For this reason, we examine and compare the localization density of two presynaptic and two postsynaptic proteins. The two presynaptic proteins we image are the vesicular protein synaptotagmin and BRP, tagged with GFP and mCherry, respectively. Both fluorescently tagged proteins are expressed through Mz19-Gal4 in a few OPNs. For the postsynaptic proteins, we image Drep-2, a regulatory synaptic protein (Andlauer et al., 2014) and the cholinergic receptor Dα7, tagged with mStrawberry and GFP, respectively. Their expression was restricted to Kenyon cells by using 201y-Gal4 fly lines.

**Figure 2:**
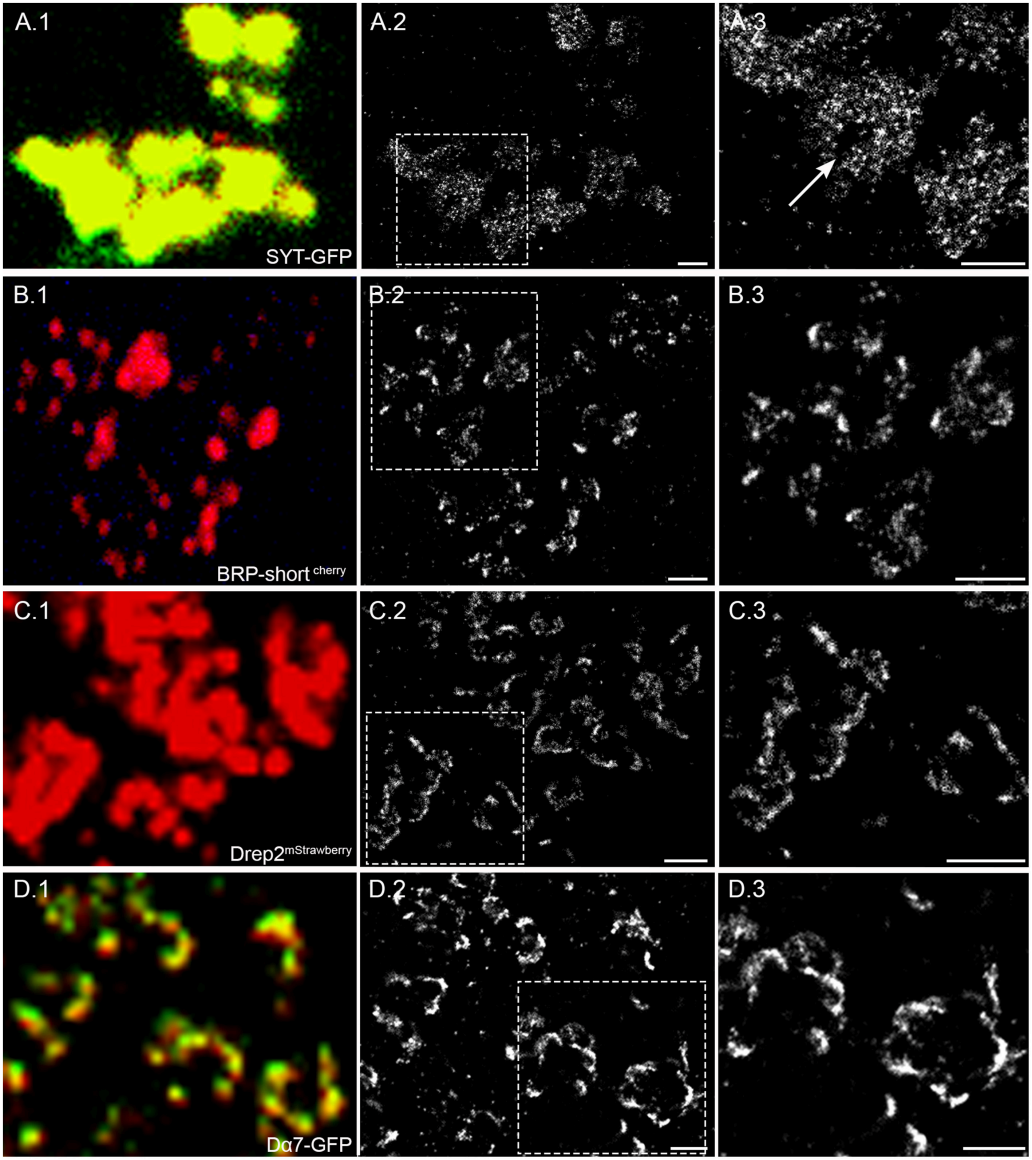
**Four different synaptic proteins imaged with confocal and super resolution microscopy**. **(A.1)** Syt-GFP expressed in OPN are related to vesicles and present in the entire bouton. The confocal image shows the Syt-GFP signal and the anti-GFP AF 647 staining. The super resolution image in **(A.2)** and an enlarged view of the framed area **(A.3)** reveal different signal densities within the bouton and also void space indicated by an arrow. **(B.1)** BRP-short^cherry^ expressed in OPNs are localized on T-bar formations on the presynaptic active zone. The confocal image shows the BRP-short^cherry^ spots additionally stained with AF 647. **(B.2)** With *d*STORM imaging the presynaptic active zone can be revealed and distinguished. **(B.3)** The enlarged view of the framed area also shows the fine detail of BRP-short^cherry^ localization within dense active zone sites and also few signals within the presynaptic membrane. **(C.1)** Drep-2^mStrawberry^ expressed specifically in KCs show the claw like shape of the dendrites. **(C.2)** In the super resolution image, the postsynaptic membrane is clearly visible and **(C.3)** confined areas of higher signal density are indicative for postsynaptic sites. **(D.1)** Dα7 tagged with GFP is expressed in dendrites of KCs and stained with AF647. **(D.2)** The super resolution images shows a high density of signals indicating potential postsynaptic sites. **(D.3)** In the enlarged view, we can observe that different neighboring sites can be separated. Scale bar = 1 μm.

Synaptotagmin (Syt) is a transmembrane protein of synaptic vesicles and plays an important role in the fusion of vesicles at presynaptic sites. Figure 2A.1 shows the Syt-GFP expression within the boutons of OPN and the additional anti-GFP staining with AF647 for *d*STORM. In the super resolution image (Figure 2A.2), we can observe signal within an area indicative of bouton sizes. In addition, there are some void areas without any signal (Figure 2A.3, arrow). As a vesicular membrane protein, we expect to see signals within the entire bouton, however some space would be taken by cell organelles excluding vesicles. From the size and shape, the void areas in the super resolution image are in agreement with mitochondria commonly present within the bouton terminals.

Figure 2B shows another example of BRP tagged with mCherry (BRP-short^cherry^) expressed in a MZ19-Gal4 fly line. Similar to Figure 1D, the location and size of active zone cannot be defined easily in the confocal image. However, we can observe clustered signals in the super resolution image (Figures 2B.2,B.3) implying T-bar formation at active zones of the OPN.

For the postsynaptic marker Drep-2^mStrawberry^, we can see in the confocal image an expression restricted to the membrane of the claw-like structured dendrites, however the single synaptic sites cannot be distinguished (Figure 2C.1). With *d*STORM imaging we can indeed observe signals along the membrane with high signal density at distinct sites (Figures 2C.2,C.3). Similar to Drep-2 the expression of Dα7-GFP also appears only on the membrane of the dendritic claws and high densities of signals are localized to confined sites in the super resolution image (Figure 2D).

In summary, we observe at the presynaptic site vesicular protein Syt distributed within the entire bouton, while BRP is localized specifically at restricted sites likely corresponding to individual T-bar formations. For the postsynaptic Drep-2 protein and cholinergic receptor Dα7, we observe signal along the membrane with spots of higher signal density indicative of postsynaptic densities.

### Quantitative image analysis reveals nanoscopic features

**Figure 3:**
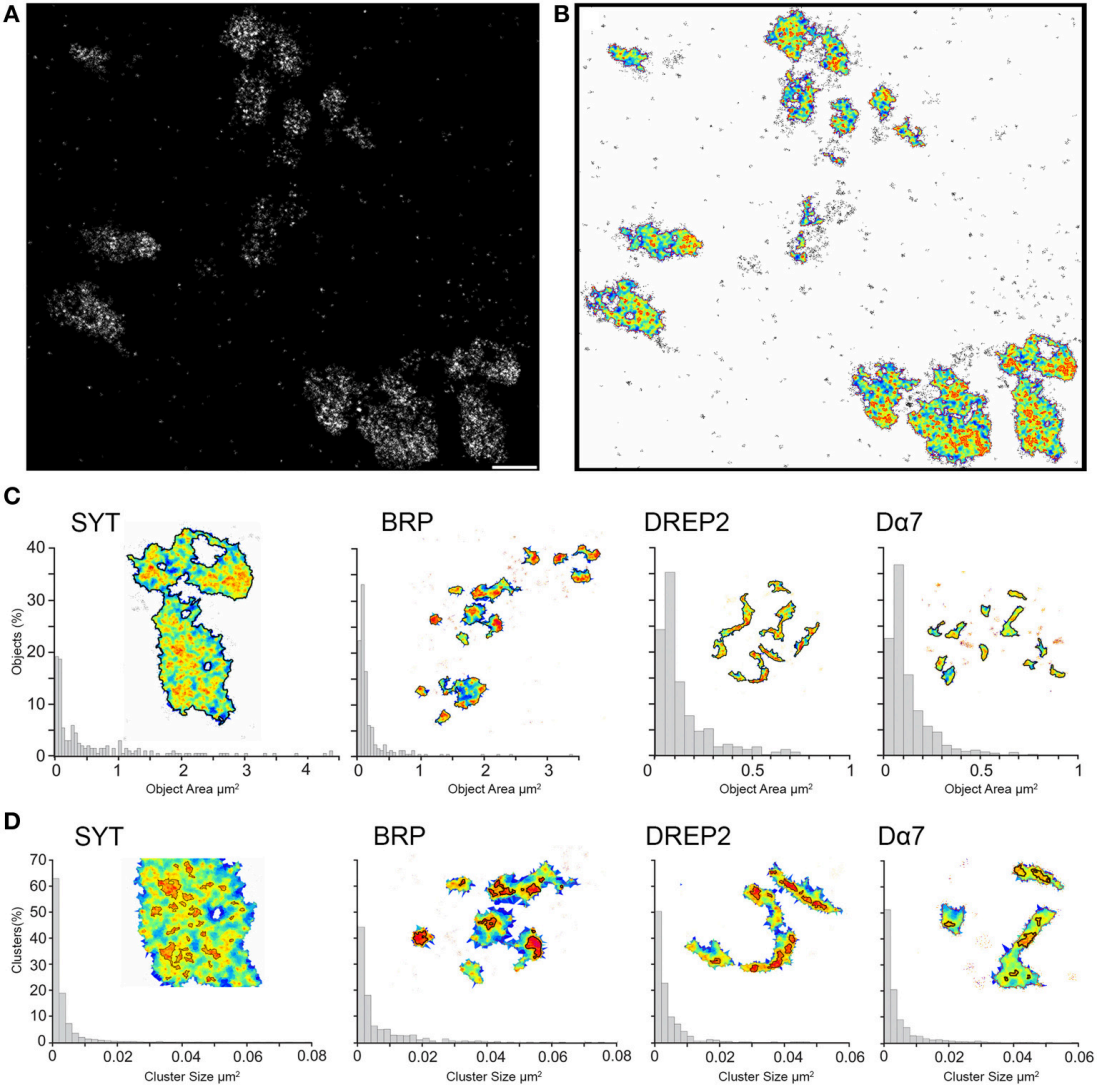
**Super resolution images segmented and analyzed for local signal density reveals objects and clusters**. **(A)** With *d*STORM we can observe in a qualitative way, different local densities of the signal related to the protein of interest, here Syt-GFP. Scale bar = 1 μm **(B)** With *SR-Tesseler* the super resolution image is segmented based on Voronoï tessellation and reveals areas with higher local densities (objects) as well as clusters within the objects. The local density is color coded in the illustration, with warmer colors indicting higher local densities. **(C)** The size distribution of object and **(D)** cluster areas of the four proteins are shown for every synaptic protein as a normalized histogram. The inset shows a close up of the structures when the threshold of the object and clusters is applied.

In order to quantitatively compare the signal densities, we investigate the super resolution images with *SR-Tesseler*, an image analysis tool based on Voronoï tessellation for image segmentation and developed specifically to examine SMLM images (Levet et al., 2015). Single molecule localizations from the super resolution images are used to determine local density so that areas of higher signal density can be revealed and quantified (Figures 3A,B). In this way areas with double the local density (objects) are defined and by setting a new threshold (1.8 times the local density) within the objects we can outline clusters. The threshold to determine objects and clusters are initially defined to exclude noise signal and for a robust object segmentation across all the samples. The same threshold has been applied to the four different synaptic proteins and we can observe that different underlying structures are outlined.

First, we analyze super resolution images of Syt-GFP boutons and obtain an average object size of 0.6 μm^2^ with values ranging from 0.03 up to 4.4 μm^2^ (*n* = 202, Figure 3C). We thus measure areas with object sizes indicative for bouton cross sections. When we apply the threshold to reveal clusters within the objects we obtain an average size of 0.003 μm^2^ (range: 0.0005–0.078 μm^2^, *n* = 2443), which corresponds to circular structures with a radius of 31 nm. This average cluster size is very close to the size of synaptic vesicles reported previously (Zhang et al., 1998). This shows that, by imaging the localization of the vesicular protein Syt-GFP, we localize indirectly the synaptic vesicles within the bouton. Clusters are found across the entire object with some domains showing higher density of signal, resulting in bigger clusters (Figure 3D). These domains are indicative of an accumulation of vesicles close to a synaptic site. Furthermore, the void spaces within the object are also clearly visible when the super resolution image is analyzed for local signal density.

With the same threshold criteria, we see for the other three synaptic proteins significantly smaller objects (*p* < 0.00001, Wilcoxon rank sum test, Figure 3C). For the presynaptic BRP we have an average object size of 0.2 μm^2^ (range: 0.03–3.36 μm^2^, *n* = 269). For the two postsynaptic proteins even smaller object areas of 0.13 μm^2^ for Drep-2 (rage: 0.03–0.74 μm^2^, *n* = 198) and 0.12 μm^2^ for Dα7 (range = 0.03–0.94 μm^2^, *n* = 863) were measured (Figure 3C). These object sizes correspond to sizes measured for synapses formed in the microglomeruli with EM (Butcher et al., 2012).

While the same threshold criteria for Syt results in cross section areas of boutons, we obtain for BRP, Drep-2 and Dα7 sizes indicative of synaptic membrane areas. When we apply subsequently the same threshold for clusters, we find an average cluster size of 0.006 μm^2^ for BRP and 0.004 μm^2^ for both Drep-2 and Dα7 (range of 0.0005–0.074/0.046/0.058 μm^2^, *n* = 841, 578, 1958, respectively, Figure 3D). BRP has significantly larger cluster size compared to all the other synaptic proteins (*p* < 0.0002), while the cluster size for Drep-2 and Dα7 are the same (*p* = 0.607). On the underlying super resolution image for the two postsynaptic proteins Drep-2 and Dα7 we observe signal along the dendritic membrane with sites of higher density. By thresholding for clusters, we would get these sites of higher protein density within the postsynaptic density. In the case of BRP thresholding for the object gives an area of BRP expression close to the presynaptic site and, when we consider the size, clusters would represent the confined and dense expression of BRP and with it a T-bar in the active zone. In this way, thresholding based on local density reveals different sizes and shapes indicative for relevant structures within the bouton, dendrite and synapse.

### 3D imaging of the synaptic protein

**Figure 4:**
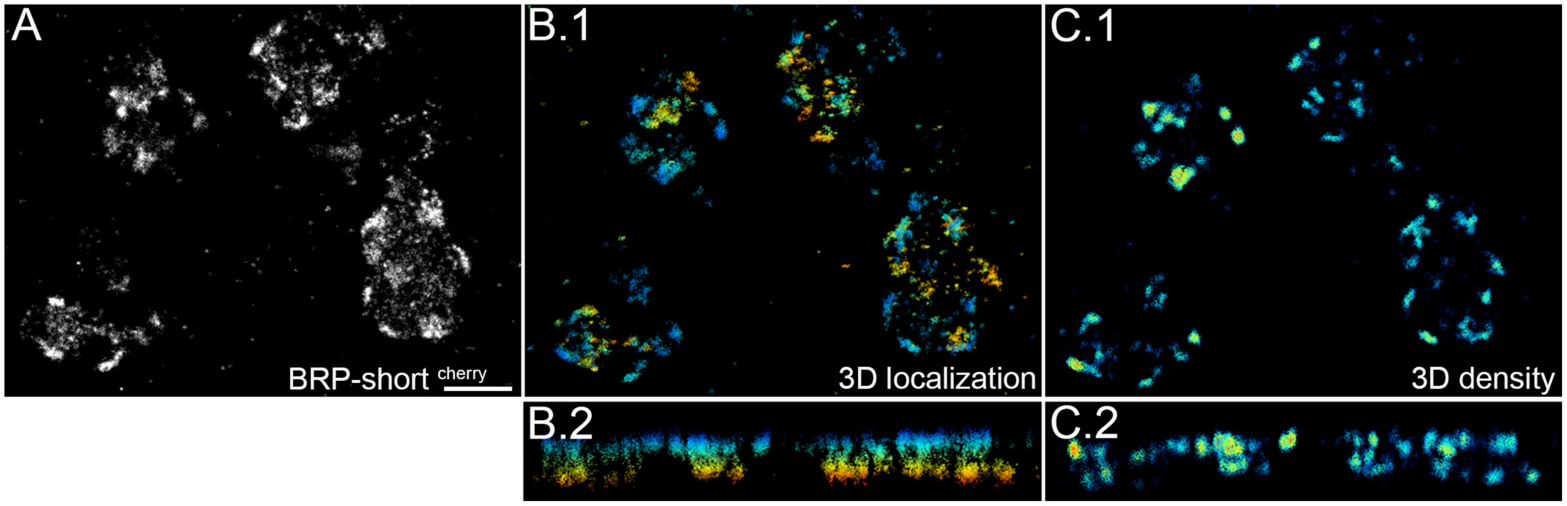
**Three dimensional super resolution image of BRP-short^cherry^**. BRP tagged with mCherry is expressed in the presynaptic active zone of OPN and imaged at a depth of 2–3 μm from the surface. **(A)** First the super resolution image is reconstructed and corrected for drift with *ThunderSTORM*. **(B.1,B.2)** Due to the illumination mode signal within a limited depth of 920 nm could be recorded and is shown color coded. **(C.1,C.2)** Density plot is calculated with a neighborhood radius of 100 nm with *ViSP* and shown color coded. We can observe that there are confined sites of similar signal density, indicative for presynaptic sites. Panels **(B.2)** and **(C.2)** show the image along the z-axis. Scale bar = 1 μm.

In the current study we focus on one-color *d*STORM in two dimensions. In order to obtain the signal localization of all three dimensions, samples should exhibit little noise, robust blinking, and the regions of interest need to be sufficiently close to the surface as we image under TIRF or highly inclined illumination conditions. 3D super resolution images are obtained by introducing astigmatism in the imaging pathway (Huang et al., 2008b). Figure 4 shows one example of the three dimensional images we have taken of BRP-short^cherry^. The imaged signals are 2–3 μm away from the surface and can be localized within a range of 1 μm. With *ViSP* the depth of the signal and the local density of signals is calculated and visualized (Figures 4B,C). The single presynaptic sites with higher BRP-expression are thus revealed within the depth of the tissue and overlapping sites in two dimensional images can easily be distinguished.

Due to the highly inclined illumination the depth of imaging is restricted and signals are localized within an optical section of 1 μm average thickness (range: 0.5–1.5 μm). Within this depth, we can image entire synaptic sites but rarely complete bouton terminals. For this reason, we get an average surface area of 13 μm^2^ and an average volume of 0.6 μm^3^ for the objects in the Syt-GFP images. In the case of BRP-short^cherry^ we get a surface area of 0.7 μm^2^ and a volume 0.03 μm^3^. Similarly, for the postsynaptic Dα7-GFP and Drep-2^mStrawberry^ we have an average surface area of 0.8 and 0.6 μm^2^ and volume of 0.04 and 0.03 μm^3^. We thus observe similar sized areas with high local density for the pre- and post-synaptic proteins expressed on the membrane of putative synaptic sites. For the vesicular protein Syt-GFP, when we consider the bigger and thus more complete objects, we obtain values for the surface area and volume in the range of bouton terminals.

To define the localization of proteins within the synapse or bouton in three dimensions adds important information especially when we want to study the relationship of different proteins expressed within a synapse. In this study, we determine the localization of specific synaptic proteins first alone and within two dimensions, as the accessible depth for SMLM is very limited. Three dimensional imaging can however reveal the relation of areas and clusters within the volume, resolve overlapping fine structures and add information about variation of signal density within a certain depth (Supplementary Movie 1).

## Discussion

The olfactory system of the fruit fly has been studied intensively, for innate odor coding but also in relation to associative memory formation (Keene and Waddell, 2007; Galizia, 2014; Hong and Luo, 2014). The antennal lobe is the first center of olfactory information processing from where OPN transmit this information either to the calyx of the mushroom body or to the lateral horn. Understanding the precise synaptic organization between distinct types of neurons is key to understand coding and decoding of olfactory information. For this purpose, recent studies combined conventional light microscopy with genetically labeled synaptic proteins in olfactory receptor neurons, local neurons, OPNs, and KCs (Kremer et al., 2010; Christiansen et al., 2011; Mosca and Luo, 2014). While these studies used a characterization of immunostained puncta based on confocal microscopy an analysis at the nanoscopic level is currently missing. In this study, we apply SMLM to tissue sections of the fly brain in order to image synapses beyond the diffraction limit and analyze the density and distribution of genetically tagged synaptic proteins expressed in few OPNs and KCs within the calyx. We show that synaptic proteins can be visualized in fine detail and compare their local density quantitatively with image segmentation and analysis methods. The SMLM approach described here will in the future allow studying changes in molecular composition of synapses and the impact of genetic manipulations in defined neurons.

### What can be resolved and revealed with *d*STORM

Super resolution microscopy provides the possibility to image beyond the diffraction limit with a significantly higher resolution as compared to conventional confocal microscopy, approaching the golden standard of EM. Moreover, multiple proteins of interest can be stained specifically with immunohistochemical methods, a methodology that is not easily combined with EM. Using the fruit fly as a model further has the advantage to visualize proteins of interest in a genetically defined type and number of neurons.

With super resolution images we can observe qualitative differences between the spatial distributions of specific proteins. To obtain a more quantitative description, we apply a recent image analysis method. Depending on the local density of our protein of interest, sizes and shapes indicative of different fine structures can be identified and measured.

In our case, the signal density related to vesicular protein synaptotagmin results in sizes and shapes of bouton cross sections and then within these bouton cross sections we observe clusters indicative of synaptic vesicles. In the super resolution images, we see how the signal related to the vesicular synaptotagmin is distributed across the entire bouton with some void areas. Based on the shape and size of the void area, we assume that they are related to cell organelles such as mitochondria that exclude vesicles. On the other hand, from domains with high signal density, we can conclude that there is an accumulation of the vesicular protein and thus vesicles what may indicate the vicinity to a presynaptic site. The other three synaptic proteins, BRP, Drep-2, and Dα7 are all localized along the membrane of the OPN bouton or claw-like dendrite of the KC. In this case, the object area is much smaller and would thus be indicative for pre- or post-synaptic densities. Thresholding within these results in clusters that could represent local nanoscopic domains of higher protein density at synaptic sites. In the case of BRP the cluster sizes are significantly larger and may represent T-bar formation of the active zone.

### Active zones imaged with confocal, electron, and super resolution microscopy

The focus of our study is on imaging and localizing synaptic proteins in the microglomeruli with *d*STORM. In order to assess whether our values are accurate, we compare them to existing anatomical studies on microglomeruli, based on confocal (Kremer et al., 2010) and electron microscopy (Butcher et al., 2012), and further on a recent study applying *d*STORM to image BRP in larval neuromuscular junction (Ehmann et al., 2014).

Kremer et al. have analyzed the same OPN expressing BRP-short^cherry^ in whole mount preparations of the fly brain using confocal imaging (Kremer et al., 2010). BRP-short^cherry^ spots were referred to as active zones, so that the area of single active zones could be estimated. With the surface tool of *Imaris* the average surface area of single BRP spots has been measured to be 3.5 μm^2^. In our study we quantify the BRP-short^cherry^ super resolution image and obtain an average object area of 0.2 μm^2^, with a range of 0.03–3.4 μm^2^. We do not look at entire stacks and only consider one 2D super resolution image. The area corresponds thus not to surface but to a cross section area. When we analyze the super resolution images of BRP-short^cherry^ in 3D we obtain an average surface area of 0.7 μm^2^ with a range of 0.15–3.4 μm^2^. With *d*STORM the individual sites of active zones can be distinguished clearly (Figure 2B) while in with confocal images very close sites may be seen as one. We attribute our measurement of smaller areas of BRP spots to the increased resolution.

The accuracy of our values can be confirmed by another anatomical study of synapses in the calyx. Butcher et al. imaged boutons of different types of OPN under the EM. They reconstructed the boutons with serial ultrathin sections and quantified the surface area, volume, and the synapses density per bouton terminal (Butcher et al., 2012). Here the size of ribbon synapses, thus synapses with T-bar formation, are 0.13 ± 0.06 μm^2^. This would correspond exactly to the average object size we obtained for BRP-short^cherry^ (0.2 μm^2^) as well as for the postsynaptic proteins Drep-2^mStrawberry^ and Dα7-GFP(0.13 μm^2^). Butcher et al. also describe that almost all synapse were divergent polyads, meaning that there is more than one postsynaptic partner for each presynaptic site. They write that the presynaptic density is often of the same size as the width of all the opposing postsynaptic elements combined. From the values we obtain from super resolution imaging we can thus confirm that there is no significant difference in the local density of signals related to BRP-short^cherry^ and the Drep-2^mStrawberry^ and Dα7-GFP, resulting in similar object sizes. However, the polyade synapse would also explain why the clusters found within the Drep-2 and Dα7 sites are smaller compared to the BRP. Having more than one postsynaptic partner implies that there is one active zone opposed to several postsynaptic partners. Clusters of higher signal within the postsynaptic site could thus represent local postsynaptic densities across the different postsynaptic partners.

Butcher et al. have further measured the surface area of bouton terminals to be on average 14–25 μm^2^, depending on the type of OPN. Here we show, from three-dimensional super resolution images, that Syt-GFP objects have on average a surface volume of 13 μm^2^ (range: 3.6–57 μm^2^) and obtain thus similar results. Taking the results from EM studies as the golden standard, we can conclude that imaging with *d*STORM gives similarly accurate results as the anatomical study based on EM images.

SMLM has not been applied to synapses in *Drosophila* brain tissue before, however, a recent study has imaged BRP in larval neuromuscular junctions (NMJ) with *d*STORM and quantified the number of proteins per active zone cytomatrix (CAZ; Ehmann et al., 2014). Despite the technical difficulties in relating localizations to number of proteins, Ehmann et al. detail a method to do so and count the number of BRP protein per CAZ and their subunits. They show that there are ~137 BRP proteins forming a unit within CAZ, composed of 15 heptametric clusters. They further show that ~26% of the signals related to BRP are not grouped into clusters. In our study, we do not quantify the protein number, as the relation of labeled molecules per localization can vary significantly depending on the antibody affinity, labeling density, and epitope accessibility across the different synaptic proteins. Instead we compare the relative densities and distributions of localizations from four different synaptic proteins to reveal cluster formations and nanoscopic patterns. Interestingly the active zone size described in the NMJ is smaller than the one observed in the OPN, however, in both cases a substantial subset of un-clustered BRP proteins were observed (Figure 2B.2). Size differences are likely due to the properties of different neuron types, in this case larval motoneurons vs. adult OPNs, or alternatively the image analysis method.

### Advantages and pitfalls of single molecule localization microscopy in tissue

In the current study, we demonstrate the potential of *d*STORM for the imaging of tissue sections of the fly brain. With the genetic possibilities in the fly system and the specific labeling of proteins, we can examine the localization and expression density of specific proteins in few defined neurons. The fly brain thus offers important advantages to minimize noise or out of focus signals. To further improve the signal to noise, we apply *d*STORM on 8 μm thin cryosections and image with highly inclined or TIRF illumination. Due to this illumination mode, we can access only structures within the first 1–3 μm. Within this depth, entire synapses can be imaged, though some may be incomplete due to sectioning or illumination angle. Further, to reconstruct several synapses along one axon or of an anatomical region such as the entire calyx is not feasible, unless one cuts serial and very thin sections (Nanguneri et al., 2012; Sigal et al., 2015). In this respect the manual labor to prepare the sample for imaging with SMLM would be comparably time consuming to EM.

Quantification of super resolution images and reliable analysis of the reconstructed signals is crucial to answer biological questions. *SR-Tesseler* allows us to perform quantitative image analysis based on local density of signals so that we can identify object of interest and clusters within the objects (Levet et al., 2015). Objects can be related to bouton cross sections or synaptic sites and clusters further reveal nanoscopic patterns of high local signal density. One needs to bear in mind that the signal we record with *d*STORM originates from the blinking of AF647 and only relates indirectly, through immunostaining, to the protein of interest. The quality of immunostaining is therefore crucial for imaging with *d*STORM and for the interpretation of the super resolution images obtained.

Though, SMLM gives more accurate measurements than confocal microscopy and similar values to those obtained with EM, one needs to consider the systematically larger sizes due to antibody staining, especially for small structures. It has been estimated that the antibodies related to the staining add ~15 nm to the actual size of the observed protein (Ehmann et al., 2014). For the large boutons and synaptic densities, the difference caused by antibody labeling is negligible. In the case of small clusters, defining the underlying structure and the geometry based on the super resolution images would require further considerations of antibody decoration and labeling density.

### Combining specific staining and highly improved resolution

Summarized, we are able to describe and map the protein organization at synapses formed between OPNs and KCs in very fine detail, beyond the diffraction limit. When we put our quantitative results in relation to what has been measured in the microglomeruli with confocal and electron microscopy, we see that with *d*STORM we obtain very similar results for bouton areas as well as sizes of synapses compared to measurements based on serial reconstructions of images taken under the EM. With SMLM, the density and distribution of proteins can further be revealed in their subcellular structures and analyzed for clusters. We can thus confirm that imaging with *d*STORM resolves the localization of proteins and can provide quantitative measures of synapse composition. With the aim to image pre- and post-synaptic proteins simultaneously, we recently started to apply *d*STORM using two different fluorophore tags.

Concluding, we believe that super resolution images will help to examine in fine detail plasticity of synapses related to density and distribution of synaptic proteins and eventually investigate its role in memory formation.

## Author contributions

FS and SS directed the project. IS performed experiments and analyzed the data with the help of GC. All authors contributed to writing the manuscript.

### Conflict of interest statement

The authors declare that the research was conducted in the absence of any commercial or financial relationships that could be construed as a potential conflict of interest.

## Supplementary material

Supplementary Movie 1 **Three dimensional super resolution microscopy of BRP-short^cherry^**. Movie shows the signal related to BRP-short^cherry^ within the volume. The localization in the z-axis is color coded. Due to the highly inclined illumination the optical section has a thickness of 920 nm. Later in the movie the density of signal within a radius of 100 nm is represented and reveals sites of higher signal density indicative for active zones in the presynaptic bouton of the OPN.

## References

[B1] Andlauer T. F. Scholz-Kornehl S. Tian R. Kirchner M. Babikir H. A. Depner H.. (2014). Drep-2 is a novel synaptic protein important for learning and memory. Elife 3:e03895. 10.7554/eLife.03895 25392983 PMC4229683

[B2] Aso Y. Hattori D. Yu Y. Johnston R. M. Iyer N. A. Ngo T. T.. (2014). The neuronal architecture of the mushroom body provides a logic for associative learning. Elife 3:e04577. 10.7554/eLife.04577 25535793 PMC4273437

[B3] Bates M. Huang B. Dempsey G. T. Zhuang X. (2007). Multicolor super-resolution imaging with photo-switchable fluorescent probes. Science 317, 1749–1753. 10.1126/science.1146598 17702910 PMC2633025

[B4] Beaudoin G. M. III Schofield C. M. Nuwal T. Zang K. Ullian E. M. Huang B.. (2012). Afadin, a Ras/Rap effector that controls cadherin function, promotes spine and excitatory synapse density in the hippocampus. J. Neurosci. 32, 99–110. 10.1523/JNEUROSCI.4565-11.2012 22219273 PMC3305287

[B5] Butcher N. J. Friedrich A. B. Lu Z. Tanimoto H. Meinertzhagen I. A. (2012). Different classes of input and output neurons reveal new features in microglomeruli of the adult *Drosophila* mushroom body calyx. J. Comp. Neurol. 520, 2185–2201. 10.1002/cne.23037 22237598

[B6] Christiansen F. Zube C. Andlauer T. F. M. Wichmann C. Fouquet W. Owald D.. (2011). Presynapses in Kenyon cell dendrites in the mushroom body calyx of *Drosophila*. J. Neurosci. 31, 9696–9707. 10.1523/JNEUROSCI.6542-10.2011 21715635 PMC6623142

[B7] Dani A. Huang B. Bergan J. Dulac C. Zhuang X. (2010). Superresolution imaging of chemical synapses in the brain. Neuron 68, 843–856. 10.1016/j.neuron.2010.11.021 21144999 PMC3057101

[B8] Dudok B. Barna L. Ledri M. Szabo S. I. Szabadits E. Pinter B.. (2015). Cell-specific STORM super-resolution imaging reveals nanoscale organization of cannabinoid signaling. Nat. Neurosci. 18, 75–86. 10.1038/nn.3892 25485758 PMC4281300

[B9] Ehmann N. Sauer M. Kittel R. J. (2015). Super-resolution microscopy of the synaptic active zone. Front. Cell. Neurosci. 9:7. 10.3389/fncel.2015.00007 25688186 PMC4311638

[B10] Ehmann N. van de Linde S. Alon A. Ljaschenko D. Keung X. Z. Holm T.. (2014). Quantitative super-resolution imaging of bruchpilot distinguishes active zone states. Nat. Commun. 5:5650. 10.1038/ncomms5650 25130366 PMC4143948

[B11] El Beheiry M. Dahan M. (2013). ViSP: representing single-particle localizations in three dimensions. Nat. Methods 10, 689–690. 10.1038/nmeth.2566 23900246

[B12] Galizia C. G. (2014). Olfactory coding in the insect brain: data and conjectures. Eur. J. Neurosci. 39, 1784–1795. 10.1111/ejn.12558 24698302 PMC4237541

[B13] Heilemann M. van de Linde S. Schuttpelz M. Kasper R. Seefeldt B. Mukherjee A.. (2008). Subdiffraction-resolution fluorescence imaging with conventional fluorescent probes. Angew. Chem. Int. Ed Engl. 47, 6172–6176. 10.1002/anie.200802376 18646237

[B14] Hong W. Luo L. (2014). Genetic control of wiring specificity in the fly olfactory system. Genetics 196, 17–29. 10.1534/genetics.113.154336 24395823 PMC3872183

[B15] Huang B. Jones S. A. Brandenburg B. Zhuang X. (2008a). Whole-cell 3D STORM reveals interactions between cellular structures with nanometer-scale resolution. Nat. Methods 5, 1047–1052. 10.1038/nmeth.1274 19029906 PMC2596623

[B16] Huang B. Wang W. Bates M. Zhuang X. (2008b). Three-dimensional super-resolution imaging by stochastic optical reconstruction microscopy. Science 319, 810–813. 10.1126/science.1153529 18174397 PMC2633023

[B17] Kamiyama D. Huang B. (2012). Development in the STORM. Dev. Cell 23, 1103–1110. 10.1016/j.devcel.2012.10.003 23237944 PMC3523271

[B18] Keene A. C. Waddell S. (2007). *Drosophila* olfactory memory: single genes to complex neural circuits. Nat. Rev. Neurosci. 8, 341–354. 10.1038/nrn2098 17453015

[B19] Kremer M. C. Christiansen F. Leiss F. Paehler M. Knapek S. Andlauer T. F.. (2010). Structural long-term changes at mushroom body input synapses. Curr. Biol. 20, 1938–1944. 10.1016/j.cub.2010.09.060 20951043

[B20] Lakadamyali M. (2012). High resolution imaging of neuronal connectivity. J. Microsc. 248, 111–116. 10.1111/j.1365-2818.2012.03638.x 22881275

[B21] Levet F. Hosy E. Kechkar A. Butler C. Beghin A. Choquet D.. (2015). SR-Tesseler: a method to segment and quantify localization-based super-resolution microscopy data. Nat. Methods 12, 1065–1071. 10.1038/nmeth.3579 26344046

[B22] Lichtman J. W. Denk W. (2011). The big and the small: challenges of imaging the brain's circuits. Science 334, 618–623. 10.1126/science.1209168 22053041

[B23] Mosca T. J. Luo L. (2014). Synaptic organization of the *Drosophila* antennal lobe and its regulation by the teneurins. Elife 3:e03726. 10.7554/eLife.03726 25310239 PMC4194450

[B24] Nanguneri S. Flottmann B. Horstmann H. Heilemann M. Kuner T. (2012). Three-dimensional, tomographic super-resolution fluorescence imaging of serially sectioned thick samples. PLoS ONE 7:e38098. 10.1371/journal.pone.0038098 22662272 PMC3360663

[B25] Ovesny M. Krizek P. Borkovec J. Svindrych Z. Hagen G. M. (2014). ThunderSTORM: a comprehensive ImageJ plug-in for PALM and STORM data analysis and super-resolution imaging. Bioinformatics 30, 2389–2390. 10.1093/bioinformatics/btu202 24771516 PMC4207427

[B26] Shih C. T. Sporns O. Yuan S. L. Su T. S. Lin Y. J. Chuang C. C.. (2015). Connectomics-based analysis of information flow in the *Drosophila* brain. Curr. Biol. 25, 1249–1258. 10.1016/j.cub.2015.03.021 25866397

[B27] Sigal Y. M. Speer C. M. Babcock H. P. Zhuang X. (2015). Mapping synaptic input fields of neurons with super-resolution imaging. Cell 163, 493–505. 10.1016/j.cell.2015.08.033 26435106 PMC4733473

[B28] Specht C. G. Izeddin I. Rodriguez P. C. El Beheiry M. Rostaing P. Darzacq X.. (2013). Quantitative nanoscopy of inhibitory synapses: counting gephyrin molecules and receptor binding sites. Neuron 79, 308–321. 10.1016/j.neuron.2013.05.013 23889935

[B29] Szymborska A. D. A. Daigle N. Cordes V. C. Briggs J. A. G. Ellenberg J. (2013). Nuclear pore scaffold structure analyzed by super-resolution microscopy and particle averaging. Science 341, 655–658. 10.1126/science.1240672 23845946

[B30] Xu K. Zhong G. Zhuang X. (2013). Actin, spectrin, and associated proteins form a periodic cytoskeletal structure in axons. Science 339, 452–456. 10.1126/science.1232251 23239625 PMC3815867

[B31] Zhang B. Young H. K. Beckstead R. B. Budnik V. Ganetzky B. Bellen H. J. (1998). Synaptic vesicle size and number are regulated by a clathrin adaptor protein required for endocytosis. Neuron 21, 1465–1475. 10.1016/S0896-6273(00)80664-9 9883738

